# Majie Cataplasm Promotes Th1 Response to Fight against Asthmatic Th2 Inflammation through NKs

**DOI:** 10.1155/2022/6745420

**Published:** 2022-05-12

**Authors:** Wenting Ji, Hanfen Shi, Tianyi Feng, Shuang Zhang, Haixia Liu, Wenxiu Xu, Xueqian Wang, Qingguo Wang

**Affiliations:** ^1^Chengdu University of Traditional Chinese Medicine, Chengdu, Sichuan 611137, China; ^2^Beijing University of Chinese Medicine, Beijing 100029, China

## Abstract

**Background:**

Immune cells are tightly bound up with the pathogenesis of asthma. Besides T cells, B cells, macrophages, and mast cells, the mechanism of innate lymphoid cells (ILCs) in asthma is gradually explicit. As a kind of traditional Chinese medicine, Majie cataplasm realizes its potential in the clinical setting as an adjuvant for asthma. In our previous experiments, Majie cataplasm inhibits the increasing Th1 and Th2 in allergic asthma inflammation and reshapes a balance between Th1 and Th2. As ILCs are the reflection of Th cells in lung tissues, we will figure out whether Majie cataplasm could have similar effects on ILCs or not.

**Methods:**

A total of 40 female C57/BL6 mice were randomly divided into the control group (*n* = 10), the asthma model group (*n* = 10), the dexamethasone group (*n* = 10), and the Majie cataplasm group (*n* = 10). Except for the control group, mice were sensitized with ovalbumin (OVA) and excited to establish mice models of asthma. Lung tissue and splenic tissue were collected at 24 h after the last challenge with OVA, and the cell suspension of the lungs and spleen was prepared. The number of ILC1s, ILC2s, ILC3s, and NKs cells in the lungs and Tregs and B10s in the spleen were detected by flow cytometry (FCM). This was followed by simultaneous quantitative detection of 40 inflammatory cytokines and chemokines in the lung by a protein microarray.

**Results:**

The dexamethasone and Majie cataplasm could restore the number of ILC1s, ILC2s, and ILC3s in lung tissue. Compared with the control group, these cells remained unchanged in the asthma model group, while ILC1s (*P* < 0.001, *P* < 0.01), ILC2s (*P* < 0.001, *P* < 0.01), and ILC3s (*P* < 0.01, *P* < 0.05) were restored after the intervention of dexamethasone and Majie cataplasm. The number of NKs was low among the control group, the asthma model group, and the dexamethasone group, while the number of NKs rocketed in the Majie cataplasm group (*P* < 0.0001). For splenic Tregs and B10s, Majie cataplasm could curb the increasing numbers of them in the asthma model group (*P* < 0.0001, *P* < 0.01), while only Tregs were suppressed by the dexamethasone (*P* < 0.0001). For the inflammatory cytokines in the lung, the contents of TNF-*α*, TNFR2, CXCL-9, CCL-12, CCL-9, CCL-2, and CCL-5 in the asthma model group were higher than those in the control group, while the contents of GM-CSF and IL-1*α* were decreased. Comparing the asthma model group to the dexamethasone group, the levels of G-CSF, CCL-9, CCL-5, and TNFR2 in the former group were higher. The levels of TNF-*α*, TNFR2, and CCL-9 in the asthma model group increase, while the levels of IFN-*γ*, IL-1*α*, ICAM-1, and IL-4 increased in the Majie cataplasm group, especially IFN-*γ* and IL-1*α*.

**Conclusion:**

Both the dexamethasone and Majie cataplasm could control the asthmatic inflammation by reducing the inflammatory factors, inhibiting the adaptive inflammation reaction in the latter stage of inflammation and furtherly reversing the inhibition of ILC2s, ILC2s, and ILC3s. In addition, Majie cataplasm can promote the quantity of NKs and the content of IL-1*α* and IFN-*γ*, induce IFN-*γ*^+^NKs to shut down the Th2 response, and tend to elicit the Th1 response.

## 1. Background

Asthma, allergic rhinitis, food allergy, and atopic eczema/dermatitis pertain to the major allergic diseases [1–3]. Nowadays, 300 million people worldwide suffer from asthma [4]. Regarding the socioeconomic impact, asthma damages the quality of life and leads to high healthcare costs [5].

Majie cataplasm, a topical drug, brings asthma patients a considerable relief. It contains *Ephedra Herba* (Mahuang) [6, 7], *Semen Sinapis* (Baijiezi) [8], *Semen Armeniacae Amarum* (Kuxingren) [9], *Rhizoma Corydalis* (Yanhusuo) [10], and *Rhizoma Zingiberis Recen*s (ginger) [11, 12]. Each of them could shape our immunity. Majie cataplasm is externally applied on the skin and is safe, convenient, and a cost-effective treatment option for clinical use [13]. We have done a lot of research and achieved the complete preparation method of Majie cataplasm, hoping that it becomes an adjuvant for asthma patients. Our clinical trial is ongoing, and according to the feedback from patients, it is found that Majie cataplasm could prevent the onset of asthma to guarantee good quality of life for patients and reduce financial burden.

Allergen-induced T helper cells 2 (Th2) have long been deemed to play a key role in asthma by inducing type 2 cytokine production in the lungs [14]. More recently, it has been getting clearer that asthma is mediated by both innate and adaptive immune cells that regulate and shape pulmonary inflammation [15]. T cells and their innate counterparts, innate lymphoid cells (ILCs), comprise the family of lymphocytes [16]. Once ILCs perceive the pathogenic tissue damage caused by a broad range of receptors for microbial products, lipid mediators, and neuronal transmitters, they are poised to secrete cytokines to shape subsequent adaptive immunity [17]. In line with the typical cytokines, phenotypes, and developmental pathways, ILCs are divided into three major groups: type 1 innate lymphoid cells (ILC1s), type 2 innate lymphoid cells (ILC2s), and type 3 innate lymphoid cells (ILC3s). Another two additional immune cell types, natural killer cells (NKs) and lymphoid tissue inducer cells (LTis), belong to ILC1s and ILC3s, respectively, because of the overlapping phenotypic, developmental, and functional properties [18]. Due to the important role of ILCs in the pathogenesis of asthma, much attention has been paid to ILCs. We, therefore, suspect Majie cataplasm may also alleviate asthma inflammation through regulating ILCs. In addition, ILCs can contact Th cells and affect their differentiation [19]. And, in our previous experiments, we found that Majie cataplasm directly regulated T helper cells 1 (Th1) and Th2. Thus, it is necessary to further figure out the distinct effects of Majie cataplasm on innate immunity and adaptive immunity.

T cells and B cells are the main participants of adaptive immunity, and both of their subpopulations exhibit cells that inhibit inflammation, namely, regulatory T cells (Tregs) and regulatory B cells (Bregs), respectively. Obviously, they are the important component of adaptive immunity. As the previous experiments focused on the effect of Majie cataplasm on the subsets of Th cells, we focus on whether this medicine has an effect on Tregs and B10s (an important subtype of Bregs). Therefore, the purpose of this study was to explore the effect of Majie cataplasm on ILCs during asthmatic inflammation. Still, combining with the effect of Majie cataplasm on Tregs and B10s, we comprehensively evaluated its regulation of innate immunity and adaptive immunity. Subsequently, we would like to shed light on a comprehensive pharmacological mechanism of this cataplasm and develop a better understanding of such external drugs of traditional Chinese medicine.

## 2. Methods

### 2.1. The Making Process of Majie Cataplasm

For more details, please refer to Supplementary Materials (available here) called “making process of Majie cataplasm.” The fixed dose of these five ingredients of Majie cataplasm is 4, 4, 4, 4, and 4 grams, respectively, (one piece per day) with 63 cm^2^ (length 9 cm ×  width 7 cm) contact area for humans. For a mouse, the area is 0.2 cm^2^. The production flow chart of Majie cataplasm was referred to our previous study [20].

### 2.2. Mice

In experiments, 6- to 10-week-old WT C57/BL6 mice were obtained from SPF Biotechnology Co., Ltd. (Beijing, China, No. SCXK 2019-0010). All mice were bred in house and kept under specific pathogen-free conditions. This study was approved by the Ethics Committee on Animal Experiments of Beijing University of Chinese Medicine (approval number: No. BUCM-4-2019031001-1077).

### 2.3. Ovalbumin (OVA)-Induced Asthma Model and Drug Intervention

According to the previous experiment [20, 21], C57 mice (*n* = 40) were divided into 4 groups, including the control group (*n* = 10), the asthma model group (*n* = 10), the dexamethasone group (*n* = 10), and the Majie cataplasm group (*n* = 10).

Except for the mice of the control group, the others received an intraperitoneal injection (i.p.) of a solution containing 0.05 mg OVA and 1 mg Alum on days 0, 7, and 14. And on days 15–25, they were challenged with intranasal injection of OVA, 2.5 mg/ml, diluted in phosphate-buffered saline (PBS), 40 *μ*l/mouse. In contrast, mice in the control group were induced the same way just with PBS.

On the fifteenth day, mice in the control group and the asthma model group were given no intervention, mice in the dexamethasone group were injected intraperitoneally with the dexamethasone sodium phosphate injection 0.1 ml/mouse (2 mg/kg) [22, 23], and mice in the Majie cataplasm group were patched with cataplasm on the mice's backs, each cataplasm staying for 24 hours [20]. After 10 days, all the mice were euthanized by cervical dislocation for the following measurements.

### 2.4. Cell Isolation

#### 2.4.1. Lung Digestion for the Isolation of Leukocytes

Lungs were prepared by chopping tissues into pieces with scissors and then digested for 45 min at 37°C in Roswell Park Memorial Institute 1640 medium (RPMI1640, Thermo Fisher Scientific, Massachusetts, USA) + 2 mg/ml Collagenase IV (Worthington, USA). The digested tissue was filtered through a 70 *μ*m strainer and washed with RPMI1640 medium (Thermo Fisher Scientific) to get the cells.

### 2.5. Cell Suspension of the Spleen

Mice spleens were finely minced and meshed through a 70 *μ*m strainer. After elimination of RBCs with an RBC lysing buffer (BD, New Jersey, USA), spleen cells were suspended in the RPMI1640 medium (Thermo Fisher Scientific).

### 2.6. Flow Cytometry

Single cell suspensions were incubated with a combination of the following fluorescently conjugated antibodies. Lineage (Lin) antibodies include markers for CD11b, CD11c, CD19, CD3*ε*, CD4, CD8a, TCR*β*, and TCR*γδ*. ILC1s are defined as CD45^+^CD27^+^CD127^+^Lin^−^ cells, ILC2s are defined as CD45^+^CDST2^+^CD127^+^Lin^−^ cells, ILC3s are defined as CD45^+^CD27^−^CD127^+^Lin^−^ cells, and NKs are defined as CD45^+^NKp46^+^CD127^−^Lin^−^ cells.

Tregs in spleen suspension were stained with CD4 and CD25. Before staining the factor forkhead box p3 (Foxp3), these cells were given fixation and permeabilization according to the protocol of intracellular staining with the Foxp3/Transcription Factor Staining Buffer Set (Thermo Fisher Scientific).

B10s in spleen suspension were stained with CD5, CD1d, and CD19. Data were acquired on a Canto II flow cytometer (BD) and analyzed using FlowJo software version 10.1 (BD, New Jersey, USA). See Table 1 for antibodies information.

### 2.7. Protein Microarray of Inflammatory Cytokines and Chemokines

The protein concentration of tissue lysates was determined using the BCA Protein Assay Kit (Thermo Fisher Scientific, Massachusetts, USA). 100 µl protein supernatant was used for cytokine and chemokine detection and quantification by a G-Series Mouse Inflammation Array 1 Kit (RayBiotech, Inc. Norcross, GA; Cat. No. GSM-INF-1). According to the manufacturer' instructions, the array was designed to quantitatively detect 40 inflammatory cytokines, chemokines, and growth factors simultaneously (Table 2). Then, the signals (Cy3 channel) were captured using the InnoScan 300 Microarray Scanner (INNOPYS, Parc d'Activités Activestre, 31 390 Carbonne, France). Quantitative data analysis was performed using RayBiotech mouse Inflammation Array 1 software (GSM-INF-1-SW Analyzer). See Table 2 for 40 inflammatory cytokines.

### 2.8. Statistical Analysis

Data are presented as mean ± standard deviation (SD). Differences between groups were analyzed by GraphPad Prism 8 software with one-way ANOVA followed by Bonferroni's multiple comparison tests. *P* < 0.05 was considered statistically significant.

## 3. Results

### 3.1. The Numbers of ILC1s, ILC2s, ILC3s, and NKs in the Lungs

**Figure 1:**
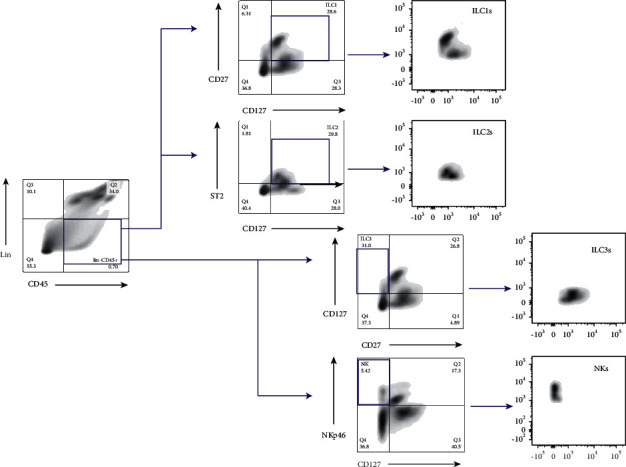
Sequential flow cytometric gating in ILC1s, ILC2s, ILC3s, and NKs in the lungs. Lineage negative gating (Lin) includes markers for CD11b, CD11c, CD19, CD3*ε*, CD4, CD8a, TCR*β*, and TCR*γδ*. ILC1s are defined as CD45^+^CD27^+^CD127^+^Lin^−^ cells, ILC2s are defined as CD45^+^ST2^+^CD127^+^Lin^−^ cells, ILC3s are defined as CD45^+^CD27^−^CD127^+^Lin^−^ cells, and NKs are defined as CD45^+^NKp46^+^CD127^−^Lin^−^ cells.

**Figure 2:**
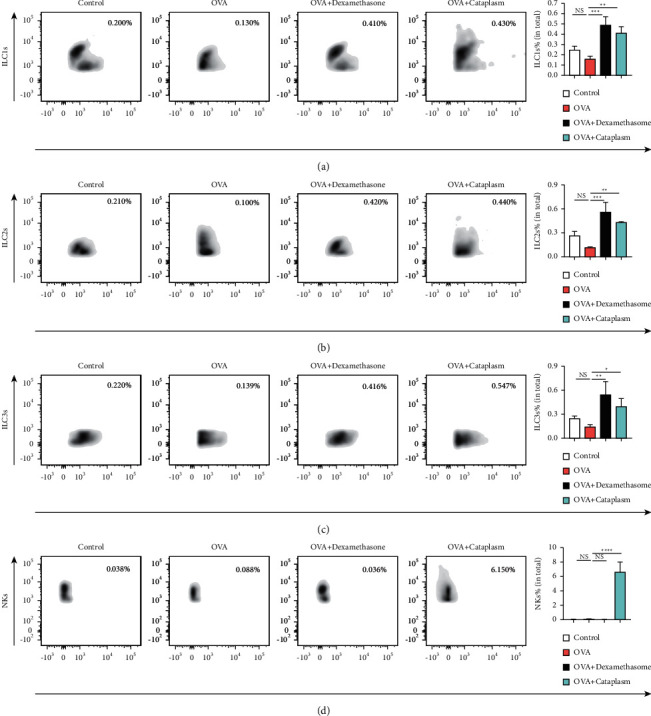
The numbers of ILC1s, ILC2s, ILC3s, and NKs in the lungs. (a–d) The results of the lungs' ILC1s, ILC2s, ILC3s, and NKs were determined by flow cytometry (numbers refer to percentages of positive cells). ^*∗*^*P* < 0.05, ^∗∗^*P* < 0.01, ^∗∗∗^*P* < 0.001, and ^∗∗∗∗^*P* < 0.0001.

We then checked the ILCs in the lungs. As shown in Figure 1, ILC1s, ILC2s, ILC3s, and NKs were circled following the sequential flow cytometric gating strategy [24]. The result of Figure 2 illustrating the numbers of ILC1s, ILC2s, ILC3s, and NKs in each group was different from our expectation. In the asthma model group, the number of ILC1s and ILC2s remained unchanged compared with the control group, while there was a marked increase in the dexamethasone group and the Majie cataplasm group (ILC1s: *P* < 0.001, *P* < 0.01; ILC2s: *P* < 0.001, *P* < 0.01). For ILC3s, the trend in results of asthma model group was consistent with that in ILC1s and ILC2s, and they also showed an increase in the dexamethasone group and the Majie cataplasm group (*P* < 0.01, *P* < 0.05). For NKs, the number varied a little and remained at a low level among the control group, the asthma model group, and the dexamethasone group. In the Majie cataplasm group, however, the number of NKs rocketed (*P* < 0.0001).

### 3.2. The Number of Tregs and B10s in Spleen Tissues

**Figure 3:**
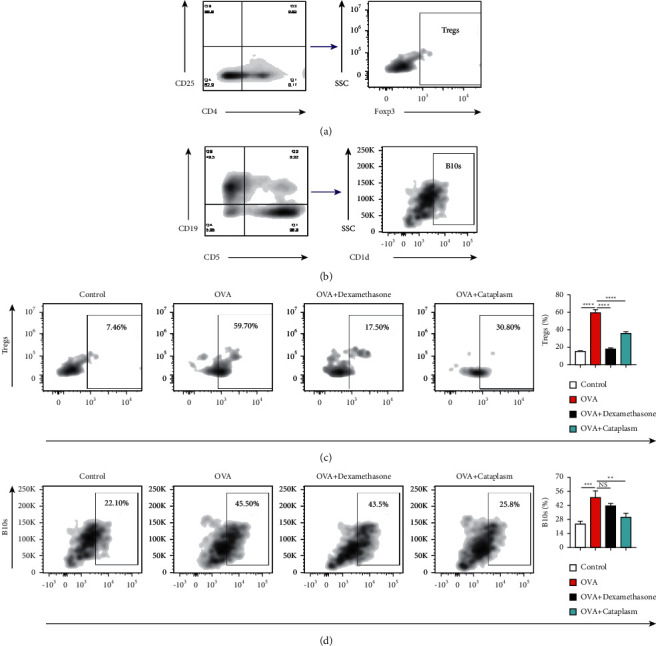
Sequential flow cytometric gating in Tregs and Bregs and the numbers of them in the spleen tissue. (a, b) The sequential flow cytometric gating in Tregs and B10s in the spleen tissue. Tregs are stained with CD4, CD25, and Foxp3. B10s were stained with CD5, CD19, and CD1d. (c, d) The result of spleen Tregs and B10s by flow cytometry (numbers refer to percentages of positive cells). ^*∗*^*P* < 0.05, ^∗∗^*P* < 0.01, ^∗∗∗^*P* < 0.001, and ^∗∗∗∗^*P* < 0.0001.

Figures 3(a) and 3(b) demonstrated the sequential flow cytometric gating strategy for Tregs and B10s, respectively. For Tregs (Figure 3(c)), compared with the control group, its quantity in the asthma model group increased dramatically (*P* < 0.0001). After the treatment of the dexamethasone and Majie cataplasm, the number of Tregs decreased remarkably (*P* < 0.0001, *P* < 0.0001). In Figure 3(d), the number of B10s owned a similar leap in the asthma model group (*P* < 0.001). The number in the dexamethasone group only showed a downward trend, yet the number fell off in the Majie cataplasm group (*P* < 0.01).

### 3.3. The Content of Inflammatory Cytokines in the Lungs

**Figure 4:**
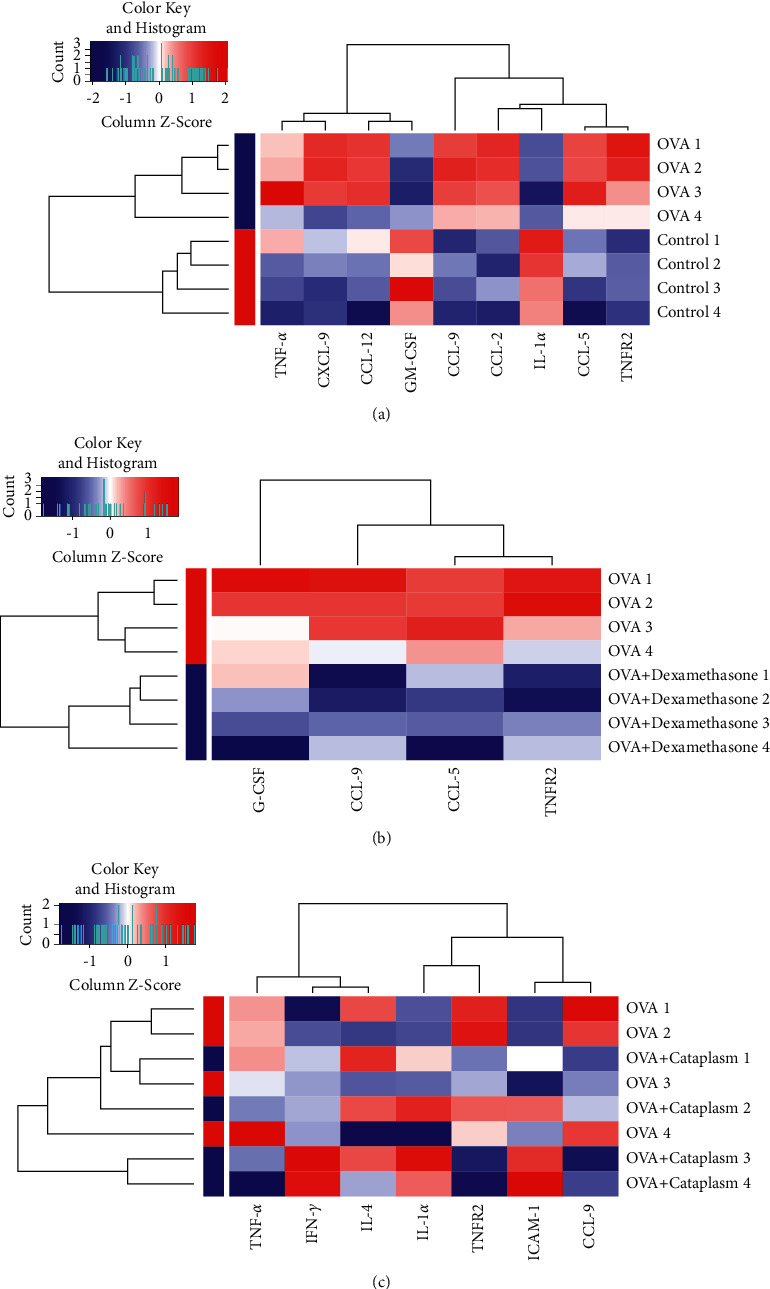
Cluster heatmaps of inflammatory cytokines/chemokines concentration of different samples in the four groups. The colorful maps were constructed by the R Project aiming to transform multiple numeric data into simplex graphical patterns. Red indicates the increased inflammatory cytokines/chemokines content, while blue indicates the opposite. And, the color intensity is proportional to the concentration of inflammatory cytokines/chemokines. (a) The differential inflammatory cytokines/chemokines between the control group (*n* = 4) and the asthma model group (*n* = 4). (b) The differential inflammatory cytokines/chemokines between the asthma model group (*n* = 4) and the dexamethasone group (*n* = 4). (c) The differential inflammatory cytokines/chemokines between the asthma model group (*n* = 4) and the Majie cataplasm group (*n* = 4).

Afterwards, we detected the inflammatory cytokines/chemokines in Figure 4. According to the results in the cluster diagram (Figure 4(a)), there were apparent differences in the expression of inflammatory factors including tumor necrosis factor alpha (TNF-*α*), chemokine C-X-C Motif Ligand 9 (CXCL-9), chemokine C-C motif ligand 12 (CCL-12), granulocyte-macrophage colony-stimulating factor (GM-CSF), chemokine C-C motif ligand 9 (CCL-9), chemokine C-C motif ligand 2 (CCL-2), interleukin 1 alpha (IL-1*α*), chemokine C-C motif ligand 5 (CCL-5), and tumor necrosis factor receptor superfamily, member 1b (TNFR2), in the asthma model group compared with the control group. Therefore, it is easy to distinguish between the two groups. In addition, the contents of TNF-*α*, TNFR2, CXCL-9, CCL-12, CCL-9, CCL-2, and CCL-5 in the asthma model group were higher than those in the control group, while the contents of GM-CSF and IL-1*α* were decreased. It could be seen from Figure 4(b) that comparing the asthma model group to the dexamethasone group, the levels of granulocyte colony stimulating factor (G-CSF), CCL-9, CCL-5, and TNFR2 in the former group were higher. We then found from Figure 4(c) that the levels of TNF-*α*, TNFR2, and CCL-9 in the asthma model group increased while the levels of interferon gamma (IFN-*γ*), IL-1*α*, intercellular adhesion molecule 1 (ICAM-1), and interleukin 4 (IL-4) increased in the Majie cataplasm group, especially IFN-*γ* and IL-1*α*.

## 4. Discussion

### 4.1. The Regeneration for ILCs

Previous studies have mainly concentrated on the function of Th2 for asthma pathogenesis, yet the focus shifts to ILCs. Usually, ILCs reside in different tissues including human blood [25], nasal polyps [26], the lungs [27], the intestine [28], and skin [29]. Given their different developments and functions, ILCs are divided into 5 subsets: NKs, ILC1s, ILC2s, ILC3s, and LTis [30].

ILC1s and NKs belong to the first group of ILCs. NKs and ILC1s have several common characteristics. Both cells secrete their main cytokine IFN-*γ* by transcription factors T-box transcription factor 21(T-bet) [31]. For ILC1s, they are generally noncytotoxic or weakly cytotoxic. NKs, in addition to the cytotoxic function, are also a key producer of type 1 cytokines, such as IFN–*γ* [32]. They express a variety of cytokine receptors, including IL-12, IL-18, IFN-*α*/-*β*, IL-2, IL-15, and IL-21 [33]. The delicate balance between activation and inhibition signals of NKs cell signals is closely associated with the growth environment of them [34]. Core cytokine of NKs as IFN-*γ* is, taking proper regulation of immune response, especially for defending against intracellular pathogens and tumors. It could be secreted by CD8^+^T cells and Th1 as well [35]. IFN-*γ* supports Th1 polarization, enhances the function of macrophages, encourages the migration of leukocyte to the infected tissues, and increases the expression of major histocompatibility complex so that T cells can better identify infected cells or malignant cells. Therefore, what counts for the balance between inflammation and immune tolerance is the regulation of IFN-*γ*. On the one hand, NKs have inevitable consequences for adaptive immunity by producing soluble factors to drive inflammation and interact with other immune cells. On the other hand, NKs is regulated by cytokines in turn. For example, on exposure to IL-12, NKs can stimulate macrophages to release IL-12 promoting T cell differentiation into Th1 [36]. Exposed to IL-4, NKs contribute to Th2 type immune response by contrast [37]. Apart from IL-12 and IL-4, NKs can also respond to other cytokines and mediate adaptive immune response. Under the impact of IFN-*γ*, NKs can activate T cells making a further increase of IFN-*γ* instead of IL-4 [38]. This means that the function of NKs for asthma is in reference to the cytokine microenvironment [39].

In view of the influence of cytokines on NKs, we detected the inflammatory factors in the lungs to make some speculation about the function of NKs to a certain extent. From the result, we can easily see that in the asthma model, the lower content of IL-1*α* is favorable for Th2 polarization, while Majie cataplasm affects NKs to reverse the shift with the increasing content of IFN-*γ* and IL-1*α*. And, another study also supported that IL-4^+^CD56^+^NKs of asthmatic patients increased in peripheral blood, yet they transformed to IFN-*γ*^+^NKs cells after drug treatment [40]. Therefore, Majie cataplasm is conducive to Th1 shift through NKs.

ILC2s share the residency with ILC1s. They secrete type 2 cytokines like IL-4, interleukin 5 (IL-5), and interleukin 13 (IL-13) [41]. Compared with other ILCs, they respond to interleukin 25 (IL-25), thymic stromal lymphopoietin (TSLP), and interleukin 33 (IL-33) and express a large number of transcription factor GATA binding protein 3 (GATA-3) which is pivotal for their development and function [27]. Extensive literature has reported that ILC2s are closely associated with airway hyperresponsiveness, eosinophilia, and mucus secretion of asthma [42].

The ILC3s and LTis are listed as the third group of ILCs because of the expression of the transcription factor ROR *γ* and the cytokine interleukin 17 (IL-17) and interleukin 22 (IL-22) [43]. Nevertheless, their developmental pathway and immune function are quite different. So far, there is few evidence to prove that LTis are involved in asthma [44].

ILC1s, ILC2s, and ILC3s are inseparable from asthma, especially the culprit of ILC2s. Therefore, we observe these cells by flow cytometry. Contrary to our prediction, the numbers of ILC1s, ILC2s, and ILC3s in the asthma group were apparently reduced, after the treatment of the dexamethasone and Majie cataplasm led to a greatly increased number of these cells. These results seem to contradict previous studies, which may be linked with the overwhelming function of adaptive immunity during a late inflammatory response. In the previous experiments, the increase of ILC2s is mostly detected in the initial stage of inflammation since stimulated with TSLP, IL-25, IL-33, and neuropeptide. In these cases, adaptive immunity is not in the prime. Once in the main period of adaptive immunity, T cell subsets (Th1, Th2, and Th17) are involved in asthma. ILC1s, ILC2s, and ILC3s, therefore, become correspondingly redundant.

### 4.2. The Control for Tregs and B10s

From the result of ILCs, we speculated that ILCs may be inhibited by T cells during the prime stage of inflammation, while dexamethasone and Majie cataplasm restored ILCs. This is because ILCs and T cells may compete with each other for their homeostasis and expansion [45]. It has been reported that there is a degree of functional redundancy of ILCs during the infection in human and mouse models [46–48]. Although ILCs have been an important source of cytokines responding to the early infection, while their function is dispensable after building on the role of adaptive immunity. And a rise in the level of the same cytokines have been found and yielded by T cell subsets. In contrast, patients of the deadly clinical condition of severe combined immunodeficiency lacking T cells and B cells demonstrate that the adaptive lymphocytes maintain nonredundant functions [49, 50]. Adaptive lymphocytes, therefore, has clearly added a selective advantage compared to that of ILCs in conferring protective immunity under natural conditions. In our previous study, Majie cataplasm could reduce the increasing number of Th1 and Th2 in the asthma model group, which meant that the adaptive cells supported the leading role in asthma inflammation [20]. And in order to confirm this, we detected other adaptive cells, Treg and B10s.

Tregs, a subset of CD4^+^T cells, has been extensively studied as they playing an important role in maintaining self-tolerance [51]. The transcription factor Foxp3 governs their function. They curb effector T cell responses by a variety of mechanisms [52] and induce strong immunosuppression.

Multiple studies in both mice and humans have demonstrated that Bregs suppress inflammatory responses chiefly via the production of interleukin 10 (IL-10) [53]. Until now, the number of Bregs subsets has been identified in mouse models. Among them, the most widely accepted subsets are CD19^+^CD5^+^CD1d^+^Bregs (also known as B10s) [54]. B10s produce IL-10 exclusively [55]. They alleviate OVA-induced allergic airway inflammation after passive transfer to sensitized recipients in an IL-10-dependent manner [56]. It is reported that the lung inflammation could be controlled when transferring IL-10^+^Bregs and IL-10^+^Tregs of OVA-induced allergic airway inflammation mice [57]. In conclusion, both B10s and Tregs have therapeutic effects on asthma.

Both of them belong to adaptive lymphocytes with a strong regulatory effect on inflammation. Our experiment found these cells played a strong antiinflammatory role in asthmatic inflammation along with increasing numbers, and they were repressed by dexamethasone and Majie cataplasm to reduce inflammatory response which was in tune with the result of Th1 and Th2 in our previous experiments [20]. Thus, adaptive lymphocytes serve in the later phase of asthmatic inflammation as they not only increase in number but also achieve their full potential on inhibiting the number and function of ILCs. The dexamethasone and Majie cataplasm facilitate the alleviation of inflammation, weaken the regulation of adaptive lymphocytes because of the reduction of Tregs and B10s, and lift restrictions on the innate immunity by recovering the number of ILC1s, ILC2s, and ILC3s. It is worth noting that the inhibition of dexamethasone on B10s is inferior to that of Majie cataplasm, implying that they have different mechanisms for defending against inflammation.

### 4.3. The Regulation for Cytokines

The result of cytokines in lung tissues could hint for asthma treatment. And, what is inconsistent with our expectation is that the content of chemokines rather than Th2 cytokines (IL-4, IL-5, IL-13, etc.) surged in the asthma model group, which indicates that the chemokines count for the pathological process of asthma. Therefore, drugs targeting chemokines may relieve asthma.

Besides, TNF-*α* released from a variety of cells (such as mast cells and macrophages) in airways [58] increased in the asthma model group. TNF-*α* multiplied in OVA-induced asthma, which not only directly promoted the inflammation of lung tissues but also enhanced ability to migrate into lung tissues and aggravate the inflammatory symptoms of asthma.

Colony stimulating factors contain macrophage colony stimulating factors M-CSF, GM-CSF, and G-CSF. Reports [59] have indicated that a rise of M-CSF and GM-CSF in the airways of patients with eosinophilic asthma facilitates allergen sensitization and airway eosinophilia in the lungs. And, G-CSF contributes to airway neutrophilia. Interestingly, in the asthma model group, GM-CSF related to the increase of eosinophils in the respiratory tract was also reduced. The drop may be caused by the inhibition of the other two GSFs and linked with the modeling method of this experiment.

The levels of M-CSF, CCL-9, CCL-5, and TNFR2 reduced after dexamethasone suppression. TNFR2 combines with TNF-*α* promoting the activation, expansion, and survival of Tregs especially in the inflammatory environment [60]. Another study demonstrated that the production of IL-10 is consistent with the expression of TNFR2 in human B cells and IL-10 could increase further through selective TNFR2 stimulation [61]. The growing number of B10s might have much to do with the increasing TNFR2.

After the intervention of Majie cataplasm, the content of TNF-*α*, TNFR2, and CCL-9 was lower than that in the asthma model group. Still, it should be noted that IFN-*γ*, IL-1*α*, ICAM-1, and IL-4 increased in the Majie cataplasm group. IL-1*α*, IFN-*γ*, and IL-12 all could induce Th1 shift [62]. Together with the increasing number of NKs detected by flow cytometry, the rising level of IFN-*γ* helps NKs develop into IFN-*γ*^+^NKs and subsequently secreting IFN-*γ*. ICAM-1 is a kind of adhesion molecule regulated by IFN-*γ* and TNF-*α*, fulfilling an important role in the recruitment of inflammatory cells. Therefore, the increase of ICAM-1 is probably caused by IFN-*γ*. IL-4 may be boosted by Majie cataplasm as the massive increase of IFN-*γ* releasing by NKs might trigger Th1 inflammation.

## 5. Conclusion

In conclusion, ILC1s, ILC2s, and ILC3s may be involved in the pathogenesis of asthma in the initial stage of inflammation and the numbers and functions of these cells are prohibited by adaptive lymphocytes including Th1, Th2, Tregs, and B10s as they serve in the later phase of asthmatic inflammation. Both the dexamethasone and Majie cataplasm cripple the inflammation with restoring numbers of ILC1s, ILC2s, and ILC3s in the lungs. In addition, Majie cataplasm could promote the proliferation of IFN-*γ*+NKs, which subsequently favors Th1 shift and inhibits Th2 migration to alleviate asthma inflammation and reshape the immune system. Instead, dexamethasone suppresses asthma well, yet it cannot reduce the recurrence of asthma for little potency in correcting the deviation of Th2 in asthmatic patients like Majie cataplasm.

## Figures and Tables

**Table 1:** Antibodies for this experiment.

Antibodies	Source	Clone
APC/Fire™ 750 anti-mouse TCR *γ*/*δ* antibody	BioLegend, USA	GL3
APC/Fire™ 750 anti-mouse CD3 antibody	BioLegend, USA	17A2
APC/Fire™ 750 anti-mouse CD4 antibody	BioLegend, USA	GK1.5
APC/Fire™ 750 anti-mouse CD8a antibody	BioLegend, USA	53–6.7
APC/Fire™ 750 anti-mouse/human CD11b antibody	BioLegend, USA	M1/70
APC/Fire™ 750 anti-mouse CD19 antibody	BioLegend, USA	6D5
APC/Fire™ 750 anti-mouse TCR *ß* chain antibody	BioLegend, USA	H57-597
APC anti-mouse CD127 (IL-7R*α*) antibody	BioLegend, USA	SB/199
FITC anti-mouse CD45 antibody	BioLegend, USA	30-F11
PerCP/Cyanine5.5 anti-mouse/rat/human CD27 antibody	BioLegend, USA	LG.3A10
PE anti-mouse CD335 (NKp46) antibody	BioLegend, USA	29A1.4
PE/Cy7 anti-mouse IL-33R*α* (ST2) antibody	BioLegend, USA	DIH4
FITC CD4 monoclonal antibody	eBioscience, USA	GK1.5
APC CD25 monoclonal antibody	eBioscience, USA	PC61.5
PE FOXP3 monoclonal antibody	eBioscience, USA	FJK-16s
APC rat anti-mouse CD5 antibody	BD Biosciences, USA	53–7.3
PE rat anti-mouse CD1d antibody	BD Biosciences, USA	1B10
FITC rat anti-mouse CD19 antibody	BD Biosciences, USA	1D3

**Table 2:** 40 inflammatory cytokines for detection.

Proinflammatory factors		IL-1*α*, IL-1*β*, IL-2, IL-3, IL-4, IL-5, IL-6, IL-7, IL-10, IL-12 p70, IL-13, IL-15, IL-17A, IL-21, IFN-*γ*
Chemokines	CXC family	CXCL1, CXCL4, CXCL-5, CXCL13, CXCL9
	CC family	CCL1, CCL2, CCL3, CCL5, CCL9, CCL11, CCL12, CCL17, CCL24
Colony stimulating factors		G-CSF, GM-CSF, M-CSF
MMPs/TIMP-1		TIMP-1
TNF-*α*		TNF-*α*, TNFR1, TNFR2, TNFSF8, FASLG
Other factors		ICAM-1, LEP

## Data Availability

The data used to support the findings of this study are available from the corresponding author upon request.

## Abbreviations

TNF-*α*: Tumor necrosis factor alpha
CXCL-9: Chemokine C-X-C motif ligand 9
CCL-12: Chemokine C-C motif ligand 12
CCL-9: Chemokine C-C motif ligand 9
CCL-5: Chemokine C-C motif ligand 5
CCL-2: Chemokine C-C motif ligand 2
IL-1*α*: Interleukin 1 alpha
TNFR2: Tumor necrosis factor receptor superfamily, member 1b
GM-CSF: Granulocyte-macrophage colony stimulating factor
G-CSF: Granulocyte colony stimulating factor
IFN-*γ*: Interferon gamma
ICAM-1: Intercellular adhesion molecule 1
IL-4: Interleukin 4
IL-5: Interleukin 5
IL-13: Interleukin 13
M-CSF: Macrophage colony stimulating factor
IL-10: Interleukin 10
IL-17: Interleukin 17
IL-22: Interleukin 22
ILCs: Innate lymphoid cells
ILC1s: Type 1 innate lymphoid cells
ILC2s: Type 2 innate lymphoid cells
ILC3s: Type 3 innate lymphoid cells
LTis: Lymphoid tissue inducer cells
NKs: Natural killer cells
Th1: T helper cells 1
Th2: T helper cells 2
Th17: T helper cells 17
Tregs: Regulatory T cells
Bregs: Regulatory B cells
B10s: CD19^+^CD5^+^CD1d^+^Bregs
Foxp3: Forkhead box P3
T-bet: T-Box transcription factor 21
COPD: Chronic obstructive pulmonary disease
GATA-3: GATA binding protein 3
RPMI1640: Roswell park memorial institute 1640 medium
OVA: Ovalbumin
PBS: Phosphate-buffered saline.

## Supplementary Materials

Supplementary Materials The material includes the process of the extraction and molding technique of Majie cataplasm. The quality evaluation methods such as TLC and HPLC are presented. It provides a detailed method and scientific evaluation for mass production of Majie cataplasm.
